# *Cnidoscolus aconitifolius* leaf pellet can manipulate rumen fermentation characteristics and nutrient degradability

**DOI:** 10.5713/ab.20.0833

**Published:** 2021-02-15

**Authors:** Pajaree Totakul, Maharach Matra, Sukruthai Sommai, Metha Wanapat

**Affiliations:** 1 Tropical Feed Resources Research and Development Center (TROFREC), Department of Animal Science, Faculty of Agriculture, Khon Kaen University, Khon Kaen 40002, Thailand

**Keywords:** Chaya Leaf, Fodder Shrub, Protein Source, Rumen Fermentation

## Abstract

**Objective:**

Chaya (*Cnidoscolus aconitifolius*) leaf has been found to be an important source of protein, vitamins, minerals, as well as phytonutrients. The present study aimed to evaluate the effect of Chaya leaf pellet (CHYP) with various level of crude protein (CP) in the concentrate on rumen fermentation characteristics and nutrient degradability in *in vitro* gas production technique.

**Methods:**

In an *in vitro* rumen fermentation study the dietary treatments were arranged according to a 3×5 factorial arrangement in a completely randomized design, consisting of Factor A: three levels of CP of concentrate mixtures (14%, 16%, and 18% CP, respectively) and Factor B: five levels of CHYP supplementation (at 0%, 2%, 4%, 6%, and 8% of dry matter substrates).

**Results:**

The gas production kinetics, fraction (a) and fraction (b) were lower (p<0.05) with an increasing CHYP addition. Additionally, the fraction (a+b) was found to yield a significant interaction (p<0.05) while the fraction (c) was not impacted by CHYP addition. However, *in vitro* DM degradability was enhanced and interactive (p<0.05), using 16% CP of concentrate with 6% and 8% CHYP, when compared with 18% CP in the non-addition. Additionally, the treatment with higher CP of the concentrate was higher in NH_3_-N concentration (p<0.001) and by CHYP supplementation group (p<0.05). Nevertheless, protozoal counts in the rumen were remarkably decreased (p<0.05) with increasing level of CHYP supplementation. Furthermore, rumen C_2_ concentration was lower (p<0.05) in the treatments with CHYP supplementation, while C_3_ was significantly increased and interactive (p<0.05) between levels of CP and CHYP supplementation especially at 8% CHYP supplementation.

**Conclusion:**

Based on this study, the results revealed CHYP as a promising feed supplement to enhance rumen fermentation and to mitigate methane production. However, *in vivo* feeding experiments should be subsequently conducted to elucidate the effect of CHYP supplementation on rumen fermentation, as well as ruminant production efficiency.

## INTRODUCTION

Ruminants play a crucial role as generators of food and income for stakeholders globally. However, these livestock systems typically possess a diet which is deficient in crude protein (CP) and are essentially high in structural carbohydrates. Without suitable supplementation, feeding such diets will result in insufficient forage intake, low feed conversion efficiency, and low animal productivity [1]. Russell and Rychlik [2] emphasized the importance of rumen ecology especially, the vital role of microbiomes on rumen fermentation efficiency and the subsequent livestock production system. Traditionally, shrubs and trees are used as feedstuff to supply livestock with energy, protein, and other nutrients [3,4]. Some of the most common trees and shrubs used in the diets have been shown to improve reproductive performance [5], body growth [6,7] and milk production [8]. The use of tree leaves in animal feeding has been practiced for a long time [9]. Fodder tree leaves are of particular importance because they contain a high level of CP, therefore, they are typically used for feeding buffaloes, beef cattle, dairy cattle, and goats. However, the potential use of an ingredient is commonly determined not only by its CP content but also by the rumen degradability, palatability, and the associative effects with other feeds in the rations [10]. In fact, Idan et al [11] reported that a higher proportion of CP in the fodder tree leaves is present in the form that is highly digestible to ruminants. Moreover, previous studies reported that feed supplement as a fodder tree mixture could enhance ruminal degradation of nutrients, volatile fatty acids (VFA), and microbial growth in the ruminants [12]. An initial *in vitro* study demonstrated that supplementation of a low-quality roughage with different kinds of tropical fodder such as *Arachis pintoi* and *Cratylia argentea* significantly enhanced rumen microbial activity and increased the extent of ruminal organic matter (OM) and fiber degradation [13]. In an *in vivo* study, Viennasay and Wanapat [14] currently reported that supplementation of fodder shrub (*Flemingia*) enhanced the nutrient digestibility, feed intake and microbial protein synthesis. Furthermore, depending on the fodder or browse species and their phytonutrients, fodder leaf supplementation could help reduce internal parasite infections [15] and methanogens [16]. The utilization of fodder and shrub leaves in ruminant feeding has been extensively investigated [1,17,18], nevertheless, some practical aspects remain to be explored.

The fodder tree leaves are highly preferred feedstuffs, since they exhibit high palatability and support the performance of livestock. A type of fodder shrub is commonly known as Chaya (*Cnidoscolus aconitifolius*). Chaya is an evergreen, insect/disease resistant and a drought-deciduous, shrub up to 6 m in height with alternate palmate lobed leaves. The leaves are large, 32 cm×30 cm, and succulent. It originated as a domesticated vegetable in the Maya region of Guatemela, Belize, South-East Mexico during pre-Cambrian period and due to its ease of cultivation and potential productivity, the plant has spread all over the world including the tropics [19,20]. Chaya can be grown year-round and it is fast growing. In Thailand, it can grow well in the hot-rainy season (May–September) [20]. Normally, it can produce 4.9 to 7.4 kg of fresh leaf per month, with annual yield ranging from 4,999 to 8,333 kg/ha. This plant has been reported to be a good source of protein with a good profile of amino acids [21] and to contain high levels of vitamins, minerals and especially phytonutrients [22]. Additionally, the phytonutrients reduced the population of the rumen protozoa. The reduction of methane producing microorganisms is reflective of the decrease in methane production [23]. Chaya leaf contains high levels of proteins and minerals, as well as having a beneficial effect on rumen fermentation, hence Chaya is a potential source of fodder to be used to improve ruminant production. Chaya appears to be a great supplement with high feed efficiency; however, there is a limited information associated with the use Chaya as a feed pellet supplementation. Therefore, the objective of this study was to investigate the potential use of Chaya as a feed pellet with different protein levels of concentrate mixture on ruminal fermentation characteristics, *in vitro* degradability, and the fermentation end-products.

## MATERIALS AND METHODS

### Animal care and management

The experiment procedure was approved by the Institute of Animals for Scientific Purpose Development (IAD), Thailand (record no. U1–06565–2526).

### Preparation of Chaya leaf pellet

After regrowth at about 4 months, Chaya leaf and young stem (CLS) were harvested from Khon Kaen province, Thailand. The harvested Chaya biomass was then chopped to 2 to 3 mm in length and then sun-dried for 2 to 3 days. Dried Chaya was ground to pass 1-mm screen size using Cyclotech Mill (Cyclotech Mill, Tecator, Hoganas, Sweden). Pellets containing 90% ground CLS, 1% molasses, 9% cassava starch, and water (Table 1) and were formulated as a pellet by Ryuzo-kun pelleting machine (Kakiuchi Co., Ltd, Nankoku, Kochi, Japan), then sundried for about 2 to 3 days and stored in the big plastic boxes for feeding.

### Experimental design, animals, and dietary substrate treatment

The 3×5 factorial arrangement in a completely randomized design (CRD) was imposed. Factor A: three ratios of CP of concentrate mixture (at 14%, 16%, and 18% CP) and Factor B: five levels of Chaya leaf pellet (CHYP) at 0%, 2%, 4%, 6%, and 8% of dry matter substrates. The CHYP, concentrate mixtures, and rice straw were oven-dried at 60°C for 48 hour and were ground through sieve 1–millimeter before using in the experiment. Ratios of roughage (R) to concentrate (C) (R:C, 70:30) was used in a concentrate mixture. Feed and chemical compositions of concentrate, rice straw and CHYP of the experiment are presented in Table 1.

The samples of concentrates, CHYP, and rice straw were separated into two separate amounts: the first part was used for DM analysis [24] and the second part was dried in the oven (60°C) for 3 to 4 days, ground to pass a 1-mm screen (Cyclotec 1093 Sample mill, Tecator, Sweden), and were analyzed for OM and CP according to AOAC [24] method. Neutral detergent fiber (NDF) and acid detergent fiber (ADF) on ash–free basis were analyzed by an Ankom fibre analyzer incubator (model no. ANKOM200, ANKOM, Fairport, NY, USA). Condensed tannins (CT) were chemically analysed by the vanillin–HCl [25] method, as modified by Wanapat and Poungchompu [26]. The total flavonoid concentration was analyzed by using the Folin–Ciocalteu reagent method [27], while minerals were analyzed using an Atomic absorption spectrometer (Model analytic jena nova 350) with the absorbance set at 425 nm spectrophotometrically (Shimadzu, Kyoto, Japan).

### Animal and preparation of rumen inoculum

Four, rumen-fistulated dairy steers with liveweight of 240±10 kg were the donors of rumen fluid. Steers received concentrate (16% CP) as a supplement at 0.5% body weight and rice straw was offered *ad libitum* for the period of at least 3 weeks. The experimental cattle were individually penned where clean fresh water and mineral blocks lick were available. Approximately 2,400 mL of the rumen fluid samples was collected before the morning feeding. The fluid was carefully filtered through four layers of cheesecloth and transferred into pre-warmed thermos flask and was then transported to the laboratory for later analyses.

### *In vitro* fermentation of substrates

Two hundred milligrams of feed samples were weighed into 60 mL serum bottles on various times of incubation with triplicates including three replications of blank. The *in vitro* gas technique procedure [28], was employed in all steps. All fermentation bottles were tightly covered with stoppers and aluminium caps and were incubated in a water bath at 39°C for 1 h before filling with 30 mL of mixed rumen inoculum [28]. The medium solution preparation and all steps were those described in detail by Blummel and Orskov [29].

### Sample collection and analysis

*Gas kinetic production*: the gas production was measured at 0, 2, 4, 6, 12, 24, 48, 72, and 96 h of incubation. Cumulative gas production data were fitted to the model of Ørskov and McDonald [30], as follow:

$$\text{Y}=\text{a}+\text{b }(1^{-\text{e}-\text{ct}})$$

Where a = the gas production from the immediately soluble fraction (mL), b = the gas production from the insoluble fraction (mL), c = the gas production rate constant for the insoluble fraction (mL/h), t = incubation time (h), a+b = the potential extent of gas production (mL), Y = gas produced at time “t” (mL).

#### In vitro dry matter digestibility (IVDMD)

Preparation and calculation were those reported by Van Soest and Robertson [31], as follows:

IVDMD=[(DM of feed taken for incubation-DM of residue)×100]/DM of feed taken for incubation

#### Determination of fermentation parameters

The rumen inoculum mixtures were sampled at 12 and 24 h of fermenting post inoculation. The pH was immediately measured using a portable pH temperature meter (HANA instruments HI 8424 microcomputer Singapore). Rumen inoculum fluid was sampled at 12 h and 24 h post inoculation. Then filtered through four layers of cheesecloth. Samples were divided into three separate parts. Rumen fluid of 18 mL were then sampled and placed in a bottle to which 2 mL of 1 M H_2_SO_4_ were added to stop the fermentation process of microbial activity and then centrifuged at 3,000×g for 10 minutes. In total, 10 mL of the supernatant portion were taken and analyzed for the VFAs by using high performance liquid chromatography [32] and rumen ammonia nitrogen (NH_3_-N) by Kjeltech Auto 1030 Analyzer [24].

The second part 1 mL of rumen fluid was collected and kept in a plastic bottle which 9 mL of 10% formalin solution was added for measuring the protozoal population using a total direct count method by haemocytometer [33]. Methane production was calculated according to Moss et al [34] was followed CH_4_ emission = 0.45 (acetate)−0.275 (propionate) +0.40 (butyrate).

### Statistical analysis

All data collected from the experiments were analyzed as a 3×5 factorial arrangement in a CRD using the PROC general linear model of SAS [35]. The statistical parameters were levels of CP, CHYP levels, and levels of CP×CHYP levels interactions. Differences among statistical treatment parameters with p<0.05 and p<0.001 were taken as significant differences.

## RESULTS

### Chemical compositions

The chemical compositions of CHYP, concentrate, and rice straw are presented in Table 1. Three concentrates mixtures were formulated to contain different levels of protein (14%, 16%, and 18% CP). The CHYP contained 90.3% DM, 23.4% CP, 19.1% NDF, 216.8% ADF, 12.7% ash, and 2.3% CT. In addition, essential amino acid profile was higher in CHYP which contained good profiles of tryptophan, phenylalanine, threonine, valine, leucine, lysine, and isoleucine. Moreover, CHYP contained 0.56% K, 4.70% Ca, 1.79% Mg, 0.71% P, 0.09% Na, and 7.6% total flavonoid. Rice straw contained 90.2% DM, 81.6% NDF, 56.2% ADF, and 15.5% ash.

### Gas production kinetics and *in vitro* dry matter digestibility

Cumulative gas production and gas production kinetics are given in Table 2. The CP level in the concentrate mixture and CHYP impacted on the immediately soluble fraction (a) and insoluble fraction (b) (p<0.001). The gas production rate constant values for the insoluble fraction ratio (c) were not found to be interactive (p>0.05). While the potential extent of gas production (a+b) was significantly interactive, as well as the cumulative gas production at 96 h. Furthermore, the CHYP supplementation factor resulted in the highest IVDMD, while the lowest was found in the non-supplementation group.

### Rumen fermentation

As shown in Table 3, the average values of rumen pH at 12 and 24 h were not changed by either the concentrate and/or by the CHYP (6.8 to 7.0). There was no interaction between NH_3_-N concentration (p>0.05) and the CP of concentrate or CHYP supplementation. Higher levels of CP of concentrate and CHYP supplementation increased the NH_3_-N concentration (p<0.001), while the protozoal count was greatly reduced (p<0.05) by the CHYP supplementation.

### Rumen volatile fatty acids concentration and methane production

The total VFA profile was influenced by high of CP of concentrate (p<0.001) and CHYP supplementation (p<0.05). Acetate (C_2_) production was found lower (p<0.05) in the treatments with CHYP supplementation. Additionally, propionate (C_3_) was significantly interactive (p<0.05) between the levels of CP of concentrate and CHYP levels, especially at 16% CP and at 6% or 8% CHYP supplementation. Furthermore, the interaction between level of CP of concentrate and the CHYP levels reduced the butyrate (C_4_), C_2_:C_3_ ratio and methane production (CH_4_) particularly, at 16% CP and with 6% and 8% CHYP inclusion (Table 4).

## DISCUSSION

### Chemical composition

Fodder tree or shrub, in particular Chaya has been reported to contain high level of CP ranging from 26% to 29% on DM basis [19,36–39]. The CP of CHYP under this experiment was 23.4% and relatively lower than previous reports. Importantly, CHYP contained 2.3% CTs and 7.6% flavonoids. Under this study, these values could be due to a number of factors namely, stage of growth, soil quality, rainfall and variety etc. Furthermore, the Chaya contained a good profile of amino acids both essential and non-essential especially methionine, lysine, tryptophan and phenylalanine etc. Nevertheless, the amino acid profile of CHYP used under this experiment was similar to those reported by other researchers [37,38,40].

### Gas production kinetics and *in vitro* dry matter digestibility

Based on this study, the gas kinetics, and immediately soluble fraction (a) were significantly different by the level of CP of concentrate. This enhanced value of gas kinetics (a) could be attributed to the higher soluble fraction. This may be due to CHYP having higher nutrients such as CP and vitamins, resulting in effective rumen fermentation. Under this study, the insoluble feed substrate (b), the gas production potential extent (a+b) was lower (p<0.001) in concentrate with 14%, 16%, and 18% CP, when the supplementation of CHYP level increased (p<0.05). As reported by Ørskov and McDonald [30] the increase in gas production (a+b) led to the decrease of the fraction (c) rate constant. Nevertheless, supplementation of CHYP impacted the cumulative gas production (at 96 h). Increasing of gas production in the present work might be due to CHYP supplementation which improved rumen fermentation and nutrient digestibility. Accordingly, Norrapoke et al [41] reported that used higher level of dietary CP resulted in an increase of gas production, *in vitro* digestibility. While in this study the IVDMD was enhanced by a high level of CP of the concentrate and by CHYP supplementation. Similarly, Anantasook and Wanapat [42] revealed that the gas production and IVDMD were linearly increased with increasing level of CP. Our results could be due to the greater IVDMD with higher level of CP and the supplementation of CHYP. This higher of CP content enhanced the nutrient digestibilities in the rumen [43]. Moreover, Woodward et al [44] stated that plants containing less than 5% CT did not impact on nutrient degradability. In this trial, the CTs in CHYP were 2.3% on the DM, hence the digestibilities of nutrients were beneficial. Similar, results were reported by Ampapon and Wanapat [45].

### Rumen fermentation

As described by Van Soest et al [46] rumen fermentation characteristics such as pH, NH3-N concentration, protozoa should be measured to determine the relationship between the diet and rumen ecology. Under this study, ruminal pH was in the range of 6.8 to 7.0 which was a normal range for rumen ecology and the efficiency of rumen fermentation. NH_3_-N concentration was increased with the supplementation of CHYP. Similarly, Promkot and Wanapat [43] reported that NH_3_-N concentration increased with higher CP content in dietary treatment. This could be due to the CHYP supplementation that enhanced rumen fermentation by providing more nutrients to the microorganisms to increase their feed degradation activities.

Furthermore, the protozoal counts were slightly decreased with increasing level of CHYP supplementation. This occurrence may be due to phytonutrients such as CT and flavonoids contained in the CHYP which reduced the protozoal population. Oskoueian et al [23] and Cushnie and Lamb [47] reported that phytonutrients may suppress the growth or activity of protozoa, through the inhibition of cytoplasmic membrane function and nucleic acid synthesis. Likewise, Rispoli et al [48] found the effect of phytonutrients significantly decreased protozoal populations in the rumen.

### Rumen volatile fatty acids concentration and methane production

Higher VFA production increased with increased levels of CP concentrate and CHYP supplementation. Hume [49] stated that higher digestibility of feeds would contribute to enhanced concentrations of VFA in the rumen. Moreover, C_2_ was decreased in the treatment with CHYP supplementation, while C_3_ was greater when using 16% CP of concentrate with 6% or 8% CHYP supplementation, while the C_2_:C_3_ ratio and CH_4_ were significantly decreased. Chen and Wolin [50] that H_2_ produced by the synthesis of C_2_ would be captured in the synthesis of C_3_ process, accordingly. As explained by Denman and McSweeney [51] methanogens are attached on the surface of protozoa in the rumen, when protozoa were decreased, hence, the methanogens would be suppressed accordingly. During rumen fermentation, 2% to 12% of ingested gross energy is changed to CH_4_, which lowers the efficiency of feed utilization [52]. Similarly, Seradj [53] reported that the supplementation of the phytonutrient Bioflavex decreased CH_4_ emission while C_3_ was increased. Additionally, Kamra [54] revealed that the phytonutrient used as feed additives would enhance the potential of rumen fermentation, reducing CH_4_ emission. Under this study, the supplementation of CHYP which contained phytonutrients decreased CH_4_ production in the rumen. Among natural plant secondary compounds, flavonoids, especially CTs and saponins, have been receiving more attention because of their wide range of biological activities and in particular, the antimicrobial properties and impacts on the rumen. Flavonoids are diverse group of phytonutrients found in almost all fruits and vegetables especially in Chaya leaf [55]. These natural compounds were shown to have direct effects against methanogens and to be an alternative agent to suppress CH_4_ production and hence, improve animal health and productivity [56].

## CONCLUSION

Based on this study, it could be concluded that supplementation of 6% or 8% CHYP with 16% CP of concentrate improved the IVDMD, enhanced the rumen C_3_, C_2_:C_3_ and mitigated the CH_4_ production. CHYP can be used as a supplemental feed to modulate the rumen fermentation and to increase feed utilization efficiency. Nevertheless, more *in vivo* work is suggested to help generate more data for further implementation.

## Figures and Tables

**Table 1:** Ingredients and chemical composition of concentrates, rice straw and *Cnidoscolus aconitifolius* leaf pellet used in the experiment

Items	Concentrates			CHYP	Rice straw

	% CP 14	16	18		
Ingredients (% as fed)					
Cassava	66	62	57	9	-
Dried brewery’s grain	10	13	15.5	-	-
Rice bran	5.5	5.5	5	-	-
Palm meal	8	8	8	-	-
Soybean meal	5	5	8	-	-
Urea	2.5	2.5	2.5	-	-
Molasses	1	1	1	1	-
Sulfur	1	1	1	-	-
Mineral mixed^1)^	1	1	1	-	-
Salt	1	1	1	-	-
Chaya leaf meal	-	-	-	90	-
Cassava powder	-	-	-	-	-
Chemical compositions					-
Dry matter	87.7	85.4	85.6	90.3	90.2
Chemical composition (% of dry matter)					
Organic matter	90.3	90.6	90.5	87.1	84.5
Ash	9.7	9.4	9.5	12.7	15.5
Crude protein	13.9	16.3	17.7	23.4	2.9
Neutral detergent fiber	16.8	20.7	21.5	19.1	81.6
Acid detergent fiber	10.4	11.1	11.6	16.8	56.2
Condensed tannins	-	-	-	2.3	-
Total flavonoids	-	-	-	7.6	-
Minerals (%)	-	-	-	-	-
Phosphorus (P)	-	-	-	0.71	-
Potassium (K)	-	-	-	0.56	-
Calcium (Ca)	-	-	-	4.70	-
Magnesium (Mg)	-	-	-	1.79	-
Sodium (Na)	-	-	-	0.09	-
Essential amino acid profile (g/kg)	-	-	-	-	-
Threonine	-	-	-	4.9	-
Methionine	-	-	-	10.8	-
Valine	-	-	-	20.4	-
Phenylalanine	-	-	-	26.6	-
Isoleucine	-	-	-	17.4	-
Leucine	-	-	-	10.6	-
Tryptophan	-	-	-	26.6	-
Lysine	-	-	-	3.2	-

CHYP, Chaya (*Cnidoscolus aconitifolious*, Mill. Johnston) leaf pellet; CP, crude protein.

1) Minerals, contains per kg: 4,000,000 IU vitamin A; 400,000 IU Vitamin D_3_; 4,000 IU vitamin E; 0.002 g vitamin B_12_; 16 g Mn; 24 g Fe; 10 g Zn; 2 g Cu; 0.05 g Se; 0.2 g Co: 0.5 g I.

**Table 2:** Effects of supplementation of *Cnidoscolus aconitifolius* leaf pellet with various levels of protein of concentrate on total gas production and *in vitro* dry matter digestibility

Concentrates	CHYP levels^1)^ (% of DM substrate)	Gas kinetics^2)^				Cumulative gas (mL) produced at 96 h	IVDMD^3)^ (%)

		a	b	c	a+b		
14% CP	0	−6.3	65.0	0.078	53.5	42.4	53.1
	2	−4.7	64.3	0.081	44.4	44.7	52.4
	4	−0.9	60.4	0.059	43.8	47.9	53.7
	6	−2.8	59.3	0.065	46.4	49.7	56.1
	8	−0.9	59.2	0.055	46.9	49.5	57.8
16% CP	0	−3.9	60.5	0.051	56.6	53.1	58.5
	2	−1.2	59.4	0.044	60.7	54.4	58.7
	4	−0.2	62.9	0.041	63.1	56.2	59.5
	6	−0.1	42.0	0.046	40.7	55.6	61.5
	8	−0.5	52.0	0.068	52.4	56.9	60.9
18% CP	0	−7.4	65.0	0.063	57.6	55.7	59.0
	2	−6.0	64.3	0.068	58.3	57.4	59.7
	4	−5.1	60.3	0.057	55.2	58.1	61.3
	6	−7.5	59.3	0.050	51.9	58.6	62.6
	8	−5.6	59.2	0.051	53.6	59.6	62.8
SEM		1.09	3.48	0.013	3.22	3.08	1.44
CP		^**^	^**^	^*^	^**^	^**^	^*^
CHYP		^**^	^**^	ns	^*^	^*^	^**^
Interaction		ns	ns	ns	^*^	^*^	ns

CHYP, *Cnidoscolus aconitifolius* leaf pellet; IVDMD, *in vitro* dry matter digestibility; CP, crude protein; SEM, standard error of mean.

1) 0%, 2%, 4%, 6%, and 8% of total substrate.

2) a, the gas production from the immediately soluble fraction; b, the gas production from the insoluble fraction; c, the gas production rate constant for the insoluble fraction ratio; a+b, the gas potential extant of gas production.

3) Average for 12 and 24 h.

ns, not significant;

* p<0.05,

** p<0.001.

**Table 3:** Effects of supplementation of *Cnidoscolus aconitifolius* leaf pellet with various levels of protein of concentrate on rumen fermentation

Concentrates	CHYP^1)^ (% of DM substrate)	pH	NH_3_-N (mg/100)	Protozoa (×10^6^ cells/mL)
14% CP	0	6.8	12.2	4.0
	2	6.9	14.3	4.2
	4	6.8	14.0	3.7
	6	7.0	14.4	3.4
	8	7.0	15.0	3.5
16% CP	0	6.8	16.7	4.1
	2	6.8	16.2	3.9
	4	6.9	17.8	3.2
	6	6.8	18.1	3.3
	8	6.8	17.6	2.8
18% CP	0	6.9	18.4	3.2
	2	7.0	18.8	3.4
	4	6.9	19.5	2.4
	6	6.9	20.3	2.8
	8	7.0	19.4	2.6
SEM		0.097	0.56	0.33
CP		ns	^**^	^**^
CHYP		ns	^**^	^*^
Interaction		ns	ns	ns

CHYP, *Cnidoscolus aconitifolius* leaf pellet; DM, dry matter; CP, crude protein; SEM, standard error of mean.

1) CHYP, Chaya leaf pellet at 0%, 2%, 4%, 6%, and 8% of total substrate.

ns, not significant;

* p<0.05,

** p<0.001.

**Table 4:** Effects of supplementation of *Cnidoscolus aconitifolius* leaf pellet with various levels of protein of concentrate on volatile fatty acids and methane production

Concentrates	CHYP^1)^	VFA (mol/100 mL)			Total VFA (mmol/L)	C_2_:C_3_^2)^	CH_4_^3)^ (mmol/L)

		C_2_	C_3_	C_4_			
14% CP	0	64.2	27.1	13.2	73.7	2.4	26.1
	2	62.7	26.3	12.3	75.0	2.3	26.3
	4	63.8	26.6	12.5	73.8	2.4	26.6
	6	65.1	28.5	10.4	75.2	2.2	25.5
	8	63.6	27.6	12.4	75.8	2.3	26.1
16% CP	0	64.2	26.6	12.7	75.4	2.4	26.4
	2	63.2	26.9	12.6	76.5	2.3	26.7
	4	63.7	26.6	12.8	77.9	2.4	26.5
	6	63.9	34.7	10.1	75.1	1.8	23.6
	8	62.6	34.5	8.9	76.5	1.7	23.3
18% CP	0	64.2	30.5	13.1	76.6	2.0	25.3
	2	62.3	31.0	12.1	79.3	2.1	23.8
	4	65.9	27.7	11.4	80.9	2.4	26.6
	6	63.7	32.6	10.3	80.8	1.9	23.9
	8	63.6	30.4	11.0	81.1	2.1	24.0
SEM		0.71	0.95	0.39	1.02	0.06	0.16
CP		ns	^**^	^*^	^**^	^**^	^**^
CHYP		^*^	^**^	^**^	^*^	^**^	^**^
Interaction		ns	^*^	^*^	ns	^**^	^**^

CHYP, *Cnidoscolus aconitifolius* leaf pellet; VFA, volatile fatty acids; CP, crude protein; SEM, standard error of mean.

1) CHYP, Chaya leaf pellet at 0%, 2%, 4%, 6%, and 8% of total substrate.

2) C_2_:C_3_, acetic acid to propionate ratio;

3) CH_4_ = (0.45×C_2_)−(0.275×C_3_)+(0.40×C_4_) (Moss et al [32]).

ns, not significant;

* p<0.05,

** p<0.001.
